# Design of an Efficient Real-Time Algorithm Using Reduced Feature Dimension for Recognition of Speed Limit Signs

**DOI:** 10.1155/2013/135614

**Published:** 2013-12-25

**Authors:** Hanmin Cho, Seungwha Han, Sun-Young Hwang

**Affiliations:** ^1^Department of Electronic Engineering, Sogang University, Seoul 121-742, Republic of Korea; ^2^Samsung Techwin R&D Center, Security Solution Division, 701 Sampyeong-dong, Bundang-gu, Seongnam-si, Gyeonggi 463-400, Republic of Korea

## Abstract

We propose a real-time algorithm for recognition of speed limit signs from a moving vehicle. Linear Discriminant Analysis (LDA) required for classification is performed by using Discrete Cosine Transform (DCT) coefficients. To reduce feature dimension in LDA, DCT coefficients are selected by a devised discriminant function derived from information obtained by training. Binarization and thinning are performed on a Region of Interest (ROI) obtained by preprocessing a detected ROI prior to DCT for further reduction of computation time in DCT. This process is performed on a sequence of image frames to increase the hit rate of recognition. Experimental results show that arithmetic operations are reduced by about 60%, while hit rates reach about 100% compared to previous works.

## 1. Introduction

Driver safety is the main concern of the advanced vehicle system which became implementable due to the development of the autonomous driving, automatic control, and imaging technology. An advanced vehicle system gives driver information related to safety by sensing the surroundings automatically [1]. Speed limit signs recognition is regarded to be helpful in safety for drivers using advanced vehicle system. The system needs to recognize the speed limit sign in the distance quickly and accurately in order to give the driver precaution in time since vehicle is moving fast. But existing algorithms perform recognition by using many features extracted from captured image, requiring a large amount of arithmetic operations for classification [2].

Several classification algorithms have been proposed, which include Neural Networks [2, 3], Support Vector Machine (SVM) [2], and Linear Discriminant Analysis (LDA) [2, 4]. Among these, SVM has relatively higher recognition rate, and LDA is used in many classification applications due to its low computational complexity. However, its computational complexity needs to be further reduced to be used in real-time application. It can be achieved by reducing the number of inputs of LDA.

This paper proposes an efficient real-time algorithm for recognition of speed limit signs by using reduced feature dimension. In this research study, DCT is employed and parts of Discrete Cosine Transform (DCT) coefficients are used as inputs to LDA instead of features extracted from image. DCT coefficients are selected by a devised discriminant function. To further reduce DCT computation time, binarization and thinning are applied to the detected Region of Interest (ROI). Image of speed limit sign in the distance obtained from camera has a low resolution and it gives poor rate of recognition. To resolve this problem, this paper proposes a recognition system using classification results on a sequence of frames. It can enhance hit rate of recognition by accumulating the probability of single frame recognition.

## 2. Background

**Figure 1:**
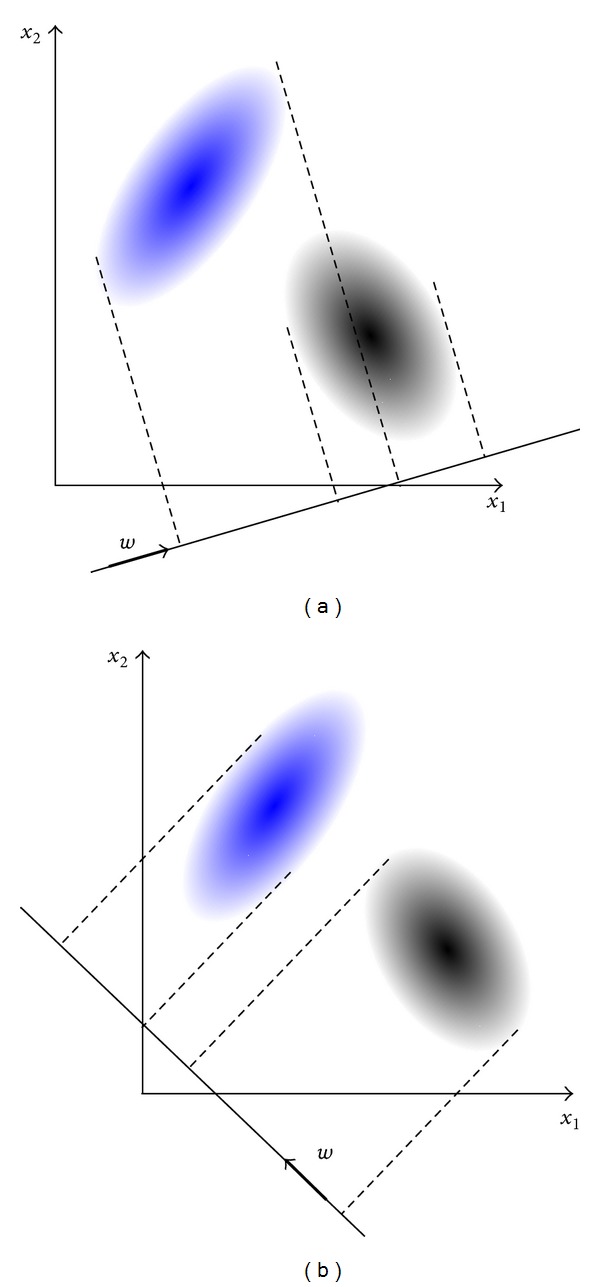
Projection of data **x** onto an axis in the direction of **w**.

In this section, LDA is briefly described, which is popularly employed for classification. LDA is a classical statistical approach for dimensionality reduction [2]. It projects high-dimensional data onto a lower dimensional space by maximizing the scatter of data points from different classes and minimizing the scatter of data belonging to the same class simultaneously, thus achieving maximum class discrimination in the dimensionality-reduced space [5]. For example, Figure 1 shows how points in 2-dimensional space can be projected onto 1-dimensional space.

The projection shown in Figure 1(b) shows more efficient separation of data than that of Figure 1(a). This concept can be expanded to *n*-dimensional space. Equations to be followed are derived to find the most efficient axis **w**. Let **x** be data points belonging to a certain class *C*_*i*_ and *y* the projection points of **x** onto axis **w**. Equation (1) shows the average of *y*, ${\tilde{m}}_{i}$, where *m*_*i*_ is mean of **x** and *n*_*i*_ is number of data:

(1) $$\begin{array}{c} {\tilde{m}}_{i}=\frac{1}{n_{i}}\underset{y\in C_{i}}{\sum }y=\frac{1}{n_{i}}\underset{x\in C_{i}}{\sum }w^{t}x=w^{t}m_{i}. \end{array}$$

It is required to find the axis **w** which maximizes the ratio of distance between ${\tilde{m}}_{1}$ and ${\tilde{m}}_{2}$ to sum of within-class scatter. This ratio can be represented as (2), where ${\tilde{s}}_{1}^{2}$, ${\tilde{s}}_{2}^{2}$ are within-class scatters of projected data in class 1 and class 2, respectively:

(2) $$\begin{array}{c} r\left(w\right)=\frac{{\left|{\tilde{m}}_{1}-{\tilde{m}}_{2}\right|}^{2}}{{\tilde{s}}_{1}^{2}+{\tilde{s}}_{2}^{2}}. \end{array}$$

Within-class scatter of class *i*, ${\tilde{s}}_{i}^{2}$, can be represented as in the following equation:

(3)   s~i2=∑x∈Ci(wtx−wtmi)2=∑x∈Ciwt(x−mi)(x−mi)tw=wtSiw.

From (3), the denominator of (2) is derived as in the following equation:

(4) $$\begin{array}{c} {\tilde{s}}_{1}^{2}+{\tilde{s}}_{2}^{2}=w^{t}\left(S_{1}+S_{2}\right)w=S_{w}. \end{array}$$

The numerator in (2), ${\left|{\tilde{m}}_{1}-{\tilde{m}}_{2}\right|}^{2}$, is shown in the following equation:

(5) |m~1−m~2|2=(wtm1−wtm2)2=wt(m1−m2)(m1−m2)tw=wtSBw.

From (4) and (5), **r**(**w**) can be written as in the following equation:

(6) $$\begin{array}{c} r\left(w\right)=\frac{w^{t}S_{B}w}{w^{t}S_{w}w}. \end{array}$$

Optimal **w**, w*, can be obtained as in the following equation, which becomes a conventional eigenvalue problem:

(7) $$\begin{array}{c} w^{*}=\arg\;\underset{w}{\max}\left\{\frac{w^{t}S_{B}w}{w^{t}S_{w}w}\right\}={S_{w}}^{-1}\left(m_{1}-m_{2}\right). \end{array}$$

Even though LDA is one of the most popular mathematical models used for classification, it is difficult to be directly used. **S**_*w*_ term in (7) becomes singular when the number of samples is much smaller than dimension of features as can be observed in many practical classification applications, which is called small sample size problem [6]. Also, high dimension of features makes LDA difficult to be directly applied to classification due to its computational complexity. To solve the problem, a method which applies Principal Component Analysis (PCA) before LDA was proposed [7, 8]. The purpose of PCA is to reduce the dimensionality while preserving the variance information as much as possible. However, it is suboptimal due to its ignorance of class information associated with patterns [9]. Direct LDA (DLDA) method [10–12] was also proposed. It directly processes data in the original high-dimensional vectors. The performance of the DLDA algorithm heavily depends on the control scheme that determines the number of features [13].

In this paper, a method which can reduce feature dimension effectively without increasing computational complexity is proposed for real-time algorithm for classification of speed limit signs.

## 3. Proposed Algorithm

As the number of operations in classification process is proportional to the number of data inputs, it is desirable to remove less significant inputs for classification [14]. By using DCT coefficients instead of features extracted from an ROI image, much less inputs are forwarded to classification process.

**Figure 2:**
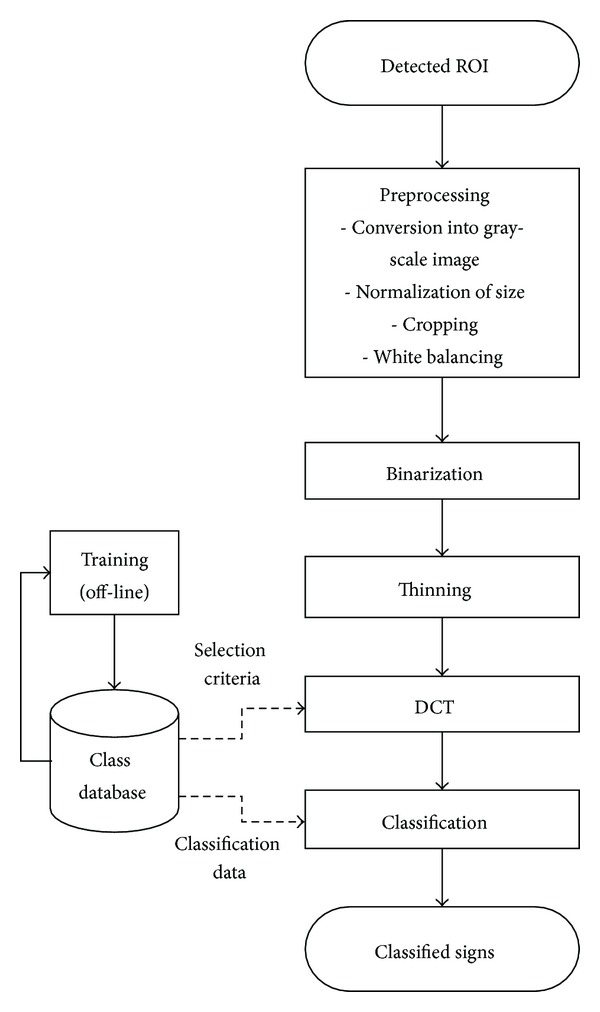
Flowchart of the proposed algorithm.

Figure 2 shows the overall flow of the proposed algorithm. After preprocessing a detected ROI, binarization and thinning are performed so that DCT computation time can be reduced. For further reduction of arithmetic operations in classification process, parts of DCT coefficients are selected by a devised discriminant function. To increase hit rate of recognition, the proposed algorithm performs classification for a sequence of images.

### 3.1. Preprocessing

**Figure 3:**
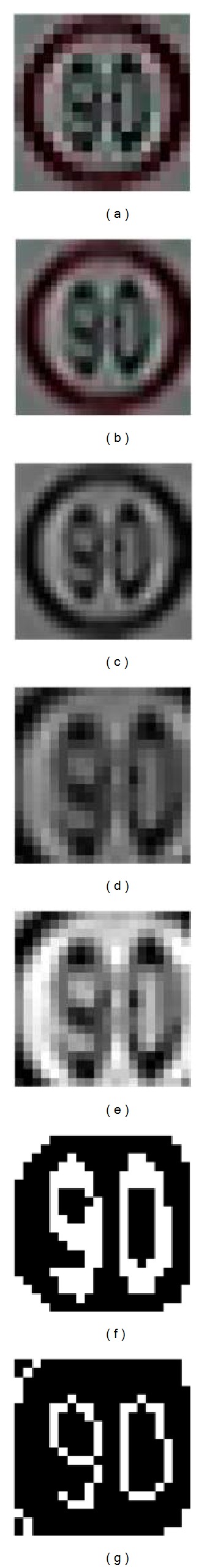
An example of ROI preprocessing in the proposed algorithm. (a) Input ROI, (b) normalized ROI, (c) gray image, (d) cropped image, (e) white balanced image, (f) binarized image, and (g) image after thinning.

Since the size of ROI varies with the distance between vehicle and speed limit sign, bicubic interpolation is employed to normalize the size of ROI into a predetermined one. Normalized ROI is converted into gray image to reduce bit width of each pixel, and the area indicating a speed limit is cropped by separating foreground from background. Then, white balancing is performed to reduce brightness variance of obtained image. To improve the resultant quality of auto white balancing, the proposed algorithm uses the white area of speed limit sign as a reference.  Figure 3 shows an example.  Figure 3(a) shows an acquired image, and Figures 3(b)–3(e) show the results of preprocessing for the image of Figure 3(a).

### 3.2. Binarization and Thinning

Prior to DCT computation, binarization and thinning are performed in the proposed algorithm. DCT computation uses each of pixel values to obtain coefficients, which require a large amount of operations for usage in real-time recognition. By using 1-bit pixels obtained by binarization, the time for multiplication can be significantly reduced. The threshold of binarization is set to 128, middle value of grayscale image, since the brightness variance has been compensated by applying white balance in advance.  Figure 3(f) shows binarized image of preprocessed ROI. Even though the feature of an image is degraded by binarization, experimental results show that the hit rate of recognition has not decreased significantly.

For further reduction of DCT computation time, thinning [15] is applied to generate more 0's in the binarized image. Thinning also removes noises remaining after binarization. The noise removal will improve classification performance. In thinning process, each pixel value is calculated by using the values of its 8 neighbors. For thinning, lookup table is used for binarized image instead of complicated operations required for gray image.  Figure 3(g) shows the image after thinning.

### 3.3. DCT Computation

2D DCT computation can be replaced by two 1D DCT computations using the row-column decomposition [16]. The time for the first 1D DCT computation can be significantly reduced due to increased number of 0-valued pixels after binarization and thinning. In the second 1D DCT computation, parts of DCT coefficients are generated, which are selected using a devised discriminant function, for reduction of computation time.

### 3.4. DCT Coefficient Selection

Classifier's performance increases dependently on the number of features. However, computational complexity and memory requirements are proportional to the number of the features both in the learning and in the classification processes. To reduce these burdens we need to remove less significant features [17]. The selected DCT coefficients are used as features in the proposed algorithm, and the performance of the classification is not degraded by using reduced amount of DCT coefficients. As mentioned in the previous section, parts of DCT coefficients are selected by using a devised discriminant function obtained through intensive analysis on the attributes of object class. The function is defined through a training process performed off-line on the classified database. The procedure to obtain discriminant function is as follows.

First, mean of DCT coefficients *D*_*c*_(*i*, *j*), *μ*_*c*_(*i*, *j*), is calculated for *N*( = 200) images per class *c*. Then, intraclass variance, intra_var_*c*_(*i*, *j*), is calculated for every *D*(*i*, *j*) by (8) and interclass variance for all the classes, inter_var(*i*, *j*) is obtained by (9):

(8) $$\begin{array}{c} {\text{intra}\_\text{var}}_{c}\left(i,j\right)=\frac{1}{N}\overset{N}{\underset{k=1}{\sum }}{\left\{D_{c}^{k}\left(i,j\right)-\mu_{c}\left(i,j\right)\right\}}^{2}, \end{array}$$

(9) $$\begin{array}{c} \text{inter}\_\text{var}\left(i,j\right)=\frac{1}{C}\overset{C}{\underset{c=1}{\sum }}{\left\{\mu_{c}\left(i,j\right)-\overset{-}{\mu_{c}\left(i,j\right)}\right\}}^{2}. \end{array}$$

Here, *D*(*i*, *j*) is coefficient of 2D DCT and *D* ^*k*^(*i*, *j*) is *D*(*i*, *j*) of the *k*th training image. From the equations above, discriminant factor for each DCT coefficient *D*(*i*, *j*) can be calculated as in the following equation:

(10) $$\begin{array}{c} \text{Discriminant}\;\;\text{Factor}\;\text{DF}\left(i,j\right)\text{=}\frac{\text{inter}\_\text{var}\left(i,j\right)}{\max_{c}\left\{{\text{intra}\_\text{var}}_{c}\left(i,j\right)\right\}}. \end{array}$$

Classification is more efficient when samples in the same class are clustered together and samples belonging to different classes are scattered in the feature space. The larger the discriminant factors are, the greater the impact on classification is in the field. The devised discriminant function selects a number of indices of 2D DCT coefficients in descending order which have large DF values. Those selected indices are used as reference positions whose corresponding DCT coefficients will be applied in classification process.

### 3.5. Classification of Speed Limit Signs

Classification is performed using the Linear Discriminant Analysis (LDA) and Mahalanobis distances [2]. LDA is performed to transform DCT coefficients into the format suitable for matching with the classes in database, and Mahalanobis distance is used as a metric for matching. Classification results for a sequence of images are used for recognition of speed limit signs. Equation (11) expresses the probability of matching with class *c* after classification for *N* image inputs:

(11) $$\begin{array}{c} P_{c}=\frac{1}{N}\overset{N}{\underset{k=1}{\sum }}\omega \left(k\right)A_{c}\left(k\right), \end{array}$$

where

(12) $$\begin{array}{c} \overset{N}{\underset{k=1}{\sum }}\omega \left(k\right)=1,\\ A_{c}\left(k\right)=\{\begin{array}{cc} 1 & \text{when}\;\arg\;\underset{c}{\min}\left({\text{MD}}_{c}\left(k\right)\right)\\ 0 & \text{otherwise}. \end{array} \end{array}$$

*ω*(*k*)'s are the weights determined experimentally. They are inversely proportional to the distance between vehicle and object. MD_*c*_(*k*) is Mahalanobis distance between captured image *k* and class *c*. The image is classified as class *c* whose probability *P*_*c*_ is the highest from *P*_1_ to *P*_*N*_.

## 4. Experimental Results

Images used for training and classification were captured on road using a mirrorless camera (MOS sensor, 4/3 inch) mounted with a 20 mm lens at 640 × 480 resolution and 30 frames/s in normal daytime. Classification is started with an image of speed limit sign captured about 30 meters away. ROI detected from the image consists of 12 × 12 pixels. We used 200 images captured at different distances for training purpose per class.  Table 1 shows the hit rates of recognition for the speed limit signs captured 1,000 times on the road.

The hit rates of recognition are about 100% when classification is performed for 7~9 consecutive images.  Table 2 compares the number of arithmetic operations with LDA and SVM [2]. The numbers in parentheses represent the reduction percentages. The numbers of arithmetic operations are reduced by about 60% and 80% when compared with LDA and SVM, respectively. In the experiments 57 DCT coefficients are selected out of 400 using the proposed discriminant function.

## 5. Conclusion

A real-time algorithm for speed limit sign recognition has been proposed with reduced amount of operations using DCT. The number of arithmetic operations was reduced by using lookup table on binarized image, which was obtained through binarization and thinning. To reduce feature dimension, discriminant function which selects parts of DCT coefficients was devised. Selection of DCT coefficients makes it possible to reduce runtime for recognition.

Accurate recognition of speed limit signs in low resolutions or in the distance is achievable by applying the proposed algorithm.

## Figures and Tables

**Table 1:** Experimental results of hit rates of recognition.

Speed (km/h)	Number of images		
	3	7	9
20	88.0%	95.7%	100.0%
30	92.0%	100.0%	100.0%
40	96.0%	97.8%	100.0%
50	92.0%	100.0%	100.0%
60	90.0%	95.6%	100.0%
70	100.0%	97.8%	100.0%
80	90.0%	100.0%	100.0%
90	98.0%	100.0%	100.0%
100	98.0%	100.0%	100.0%
110	94.0%	100.0%	100.0%

Average	93.8%	98.7%	100.0%

**Table 2:** Experimental results of number of arithmetic operations.

Operations	Methods		
	LDA	SVM	Proposed (comparison)
Add	4,000	9,697	1,570 (−60.7%/−83.8%)
Multiplication	3,990	7,297	1,731 (−56.6%/−76.2%)
